# A model to predict the function of hypothetical proteins through a nine-point classification scoring schema

**DOI:** 10.1186/s12859-018-2554-y

**Published:** 2019-01-08

**Authors:** Johny Ijaq, Girik Malik, Anuj Kumar, Partha Sarathi Das, Narendra Meena, Neeraja Bethi, Vijayaraghava Seshadri Sundararajan, Prashanth Suravajhala

**Affiliations:** 1 Department of Biotechnology, Osmania University, Hyderabad, 500007 India; 2 Department of Pediatrics, The Battelle Center for Mathematical Medicine, The Research Institute at Nationwide Children’s Hospital, The Ohio State University, Columbus, OH USA; 3 Bioclues.org, Kukatpally, Hyderabad, 500072 India; 4 Advanced Center for Computational and Applied Biotechnology, Uttarakhand Council for Biotechnology, Dehradun, 248007 India; 5 Department of Microbiology, Bioinformatics Infrastructure Facility, Vidyasagar University, Midnapore, India; 6 Department of Biotechnology and Bioinformatics, Birla Institute of Scientific Research, Statue Circle, RJ 302001 India; 7 Labrynthe, New Delhi, India

**Keywords:** Hypothetical proteins, Machine learning, Classification features, Functional genomics

## Abstract

**Background:**

Hypothetical proteins [HP] are those that are predicted to be expressed in an organism, but no evidence of their existence is known. In the recent past, annotation and curation efforts have helped overcome the challenge in understanding their diverse functions. Techniques to decipher sequence-structure-function relationship, especially in terms of functional modelling of the HPs have been developed by researchers, but using the features as classifiers for HPs has not been attempted. With the rise in number of annotation strategies, next-generation sequencing methods have provided further understanding the functions of HPs.

**Results:**

In our previous work, we developed a six-point classification scoring schema with annotation pertaining to protein family scores, orthology, protein interaction/association studies, bidirectional best BLAST hits, sorting signals, known databases and visualizers which were used to validate protein interactions. In this study, we introduced three more classifiers to our annotation system, viz. pseudogenes linked to HPs, homology modelling and non-coding RNAs associated to HPs. We discuss the challenges and performance of these classifiers using machine learning heuristics with an improved accuracy from Perceptron (81.08 to 97.67), Naive Bayes (54.05 to 96.67), Decision tree J48 (67.57 to 97.00), and SMO_npolyk (59.46 to 96.67).

**Conclusion:**

With the introduction of three new classification features, the performance of the nine-point classification scoring schema has an improved accuracy to functionally annotate the HPs.

**Electronic supplementary material:**

The online version of this article (10.1186/s12859-018-2554-y) contains supplementary material, which is available to authorized users.

## Background

Proteins that are predicted to be expressed from an open reading frame, but for which there is no experimental evidence of translation are known as hypothetical proteins (HPs). Across the whole genome, approximately 2% of the genes code for proteins, while the remaining are non-coding or still functionally unknown [1]. These known-unknown regions for which no functional links are discovered, i.e. those with no biochemical properties or obvious relatives in protein and nucleic acid databases are known as orphan genes, and the end products are called HPs [2]. These proteins are of great importance, as many of them might be associated with human diseases, thus falling into functional families. Despite their lack of functional characterization, they play an important role in understanding biochemical and physiological pathways; for example, in finding new structures and functions [3], markers and pharmacological targets [4] and early detection and benefits for proteomic and genomic research [5]. In the recent past, many efficient approaches have existed and the tools are publicly available to predict the function of the HPs. One such widely used technique is protein-protein interaction (PPI) analyses, which is considered valuable in interpreting the function of HPs [6]. While many proteins often interact with other proteins towards expediting their functions, there are challenges that are not just limited to their function but also to their regulation [7]. Therefore, characterizing the uncharacterized proteins helps to understand the biological architecture of the cell [8]. While high-throughput experimental methods like the yeast two-hybrid (Y2H) method and mass spectrometry are available to discern the function of proteins, the datasets generated by these methods tend to be incomplete and generate false positives [9]. Along with PPIs, there are other methods to identify the essentiality of proteins, such as antisense RNA [10], RNA interference [11], single-gene deletions [12] and transposon mutagenesis [13]. However, all these approaches are tedious, expensive and laborious; therefore, computational approaches combined with high-throughput experimental datasets are required to identify the function of proteins [9, 14]. Different computational methods have been designed for estimating protein function based on the information generated from sequence similarity, subcellular localization, phylogenetic profiles, mRNA expression profiles, homology modelling etc. [15]. Very recently, Lei et al. predicted essential proteins based on RNA-Seq, subcellular localization and GO annotation datasets [16, 17]. Furthermore, tools such as “LOCALIZER” [18], that predicts subcellular localization of both plant and effector proteins in the plant cell, and IncLocator [19] have been useful in predicting subcellular localization for long non-coding RNAs based on stacked ensemble classifiers [19]. On the other hand, combined analysis of all these methods or datasets is considered to be more predictive in integrating heterogeneous biological datasets [9]. Genome-wide expression analysis, machine learning, data mining, deep learning and Markov random fields are the other prediction methods which are widely employed [20, 21], whereas Support Vector Machines (SVM) [22], Neural Networks [23], Bayesian Networks [24, 25], Probabilistic Decision Trees [26], Rosetta Stone [14, 27], Gene Clustering and Network Neighbourhood analyses [28] have been used to combine different biological data sources to interpret biological relationships. Although these have shown to be successful in predicting protein function, annotation based on feature selection for inferring the function of HPs is wanting. Nevertheless, there has been a steady increase in the use of imparting machine learning and information theoretic features used for development of efficient framework for predicting interactions between proteins [28–30].

In this paper, we present a machine learning based approach to predict whether or not the given HP is functional. This method is not based on homology comparison to experimentally verified essential genes, but depends on the sequence-, topological- and Structure-based features that correlate with protein essentiality at the gene level. Features are the observable quantities that are given as input to a machine learning algorithm. Data given across each feature is used by the learning algorithm to predict the output variables. Therefore, selecting the relevant features that could predict the desired outputs is important. There are various features that define the essentiality of the proteins. In our previous study [31], we selected six such features (orthology mapping, back-to-back orthology, domain analysis, sorting signals and sub-cellular localization, functional linkages, and protein interactions) that are potentially viable to predict the function of HPs. Although the prediction performance of the selected features was shown to be acceptable, in this present study we added data on pseudogenes, non-coding RNA and homology modelling to increase the predictability of functionality of these known-unknowns. The additional features which we employed are extended to show the possibility of pseudogenes linked to HPs, proteins that are essentially structural ‘mers’ of the candidate proteins and presence of non-coding RNA signatures. We discuss the performance of newly introduced classification features from a machine learning perspective to validate the function of HPs.

## Results

We report the improved classification efficiency when three additional features were introduced (Table 1) to our earlier proposed six-point classification scoring schema. When we analysed the data through 10-fold cross-validation using the WEKA machine learning package, the decision trees (J48) yielded an accuracy of 97%, with SVM (SMO) performing high: 98, 93, 96 for Poly, RBF, npolyk kernals respectively; MLP (neural network perceptron) with 97.67% and Naive Baiyes multinomial with 98.33% (Table 2). Among the classifiers that we evaluated using WEKA, neural networks yielded the best performance with a steady change in performance of the model. In addition, one-way ANOVA with significance level (α) of 0.05 was performed to ascertain the statistical significance of the mean differences across the columns or groups based on the *p*-value. The results were found to be statistically significant and in agreement with p-value heuristics (positive and negative p-value of 3.166E-290 and 0, respectively). To check the similarity and diversity of the samples, Jaccard index similarity coefficient was plotted, providing different values ranging from perfect similarity (value 1) to low similarity (threshold value). This was further augmented when we compared the HPs from underlying similarity/distance matrix scores for evaluation. Furthermore, Jaccard index statistics revealed that the HPs annotated are inferential with the first six classifiers, but the newly introduced classifiers tend to fall apart with the introduction of non-coding elements (more details in Additional file 1: Figure S2). Secondly, the negative dataset, which we call a discrete dataset, is in principle a list of all known proteins from GenBank falling under important types of HPs. The 194 proteins are probably scaled to only these types, generating bias with the rest of the features. Thus, we argue that the negative dataset was largely more discrete and would have a more stringent heuristic learning set. To further check the redundancy, a pocket variant of perceptron algorithm was used as a unit step function, starting with a random w’ (weight) vector of length 9, eta (positive scale factor) as 0.2 and n as 1000. Invariably, perceptron gave better validation across all classifiers. For example, with a random split of 66% for the training and testing set, after 1000 iterations we obtained an average accuracy of 94.04%, with a maximum 97.97% and a minimum of 60.60%. The split performed was found to be random from all iterations, with no data point from the learning set being used in the testing set. While the SVM yielded an average accuracy of 97.36%, with a max of 100% and min of 88.13%, Naive Bayes, on the other hand, gave an accuracy of 96.62%, with a max of 100% and a min of 88.13%.

**Table 1:** Description of annotation for the three newly introduced features

Feature	Principle	Scoring criteria	Result
*Pseudogenes linked to HPs*	It is generally believed that the majority of HPs are the products of pseudogenes. Follow-up of BLAST: if the hits do not have starting codon ATG across six reading frames, then it may be assumed to be a pseudogene.	Predicted and synthetic sequences, sequences with end-to-end alignment are ignored. Sequences from *Homo sapiens* with E- value less than zero are considered.	Sequences starting without methionine and meeting all the above criteria were given 1, otherwise 0.
*Homology Modelling*	As sequence-structure implies function, it is possible to assign function to HP if we could model the protein to find any interacting domains.	Based on % identity between query and PDB template	If there is more than 30% similarity, score = 1, otherwise 0.
*Non-coding RNAs associated to HPs*	Most of the HPs from GenBank lack protein coding capacity and some of them may themselves be noncoding RNAs	The top three hits are considered for sequences from *Homo sapiens*, while the top five hits are considered when there is no considerable difference between scores.	If the above criterion is met, score 1, otherwise 0.

**Table 2:** Comparison of all accuracies of all features using multiple learning algorithms derived through WEKA (ver 3.8) with additional 3 new features increasing accuracy of the model

Learning algorithms	Accuracy with all 9 features	Average accuracy	Accuracy with all 6 features
trees_j48	97.00	95.85	67.57
trees_DecisionStump	86.33		45.95
trees_RandomForest	98.00		70.27
trees_REPTree	98.00		43.24
HoeffdingTree	96.67		Not reported
trees_LMT	98.33		70.27
trees_RandomTree	96.67		67.57
functions_smo_PolyK	98.33	96.33	78.38
functions_smo_RBFK	93.00		24.32
functions_smo_npolyk	96.67		59.46
functions_smo_Puk	97.33		Not reported
functions_RBFNetwork	96.67	97.11	48.65
functions_mlp	97.67		81.08
functions_VotedPerceptron	97.00		Not reported
bayes_nbay	96.67	94.83	54.05
bayes_NaiveBayesUpdateable	96.67		55.21
bayes_NaiveBayesMultinomial	93.00		Not reported
bayes_NaiveBayesMultinomialUpdateable	93.00		Not reported

## Discussion

The statistical evaluation suggests that among the newly introduced classifiers, non-coding RNAs and pseudogene features show considerable impact, indicating that most of the HPs are either the products of pseudogenes or linked to ncRNAs (Table 3). Among the other six features, functional linkages, pfam and orthology are highly significant, indicating that annotating the HPs across these features would predict the probable function of HPs (Table 3). Feature selection algorithms like Correlation-based Feature Selection (CFS) and Principal Component Analysis (PCA) also showed improved accuracy, whereas the accuracies on the entire data (ALL) are highest among the three methods indicating the importance of all the nine features in model generation (Table 4). In addition, we derived the best data subsets from the nine features by selecting top scores from all combinations with an ALL subset combination method “1 2 4 6 7 9” by functions_mlp (98.33) and PCA selected data subset “1 2 3 4 5 6 7 8” by functions_smo_npolyk (97.00) and trees_j48 (97.00) as the best accuracies (Table 5).

**Table 3:** Ranking to show the impact of each feature (Rank 1: High impact, Rank 9: Less impact)

Features	Functions_ smo_npolyk	trees_ j48	bayes_ nbay	Functions_mlp	Rules NNge
Pfam	5	5	5	5	5
Orthology	4	4	4	4	4
Pro_intercations	6	6	6	6	9
Bidirectional_best_blast_hits	7	7	7	7	8
Subcellular_location	7	7	7	9	7
Functional_linkages	2	2	2	2	3
Pseudogenes	3	3	3	3	1
Homology modelling	7	7	7	7	6
Non-coding RNAs	1	1	1	1	2

**Table 4:** Derived accuracies by learning algorithms with default parameters set by WEKA are listed above. Column 1 lists different algorithms

Algorithms	ALL		Cfs		PCA	
	Earlier study [25]	Current study	Earlier study [25]	Current study	Earlier study [25]	Current study
Selected Features □	1,2,3,4,5,6	1,2,3,4,5,6,7,8,9	1 2 5 6	1,2,3,6,7,9	1,2,3,4,5,6	1,2,3,4,5,6,7,8
bayes_NaiveBayesUpdateable	55.21	96.67	54.05	96.67	72.97	93.00
functions_smo_npolyk	59.46	96.67	54.05	96.00	51.35	97.00
rules_DecisionTable	48.65	96.00	54.05	96.00	70.27	92.33
functions_mlp	81.08	97.67	59.46	96.67	81.08	96.00
bayes_nbay	54.05	96.67	54.05	96.67	72.97	93.00
trees_j48	67.57	97.00	51.35	96.00	72.97	97.00
Average		97.39		96.26		94.53

Column 2 shows accuracies on the entire data through ten-fold cross-validation. Columns 3 and 4 show accuracies by different algorithms after applying feature selection algorithms as per the column header (*Cfs* Correlation Feature Selection, *PCA* Principal Component Analysis). Cfs uses best fit method and PCA uses Ranker method as set by WEKA

**Table 5:** Subset evaluation. Accuracies by learning algorithms with default parameters set by WEKA and best data subset by combination (Column 3) and Feature selection method (column 5) are listed above

Algorithms	Best combination Subsets (from complete dataset)	Accuracy	Feature selection subsets	Accuracy
bayes_NaiveBayesUpdateable	1,6,7,9	96.67	Cfs 1,2,3,6,7,9	96.67
functions_smo_npolyk	1,2,4,6,7,9	98.00	PCA 1,2,3,4,5,6,7,8	97.00
rules_DecisionTable	6,7,9	96.00	Cfs 1,2,3,6,7,9	96.00
functions_mlp	1,2,4,6,7,9	98.33	Cfs 1,2,3,6,7,9	96.67
bayes_nbay	1,6,7,9	96.67	Cfs 1,2,3,6,7,9	96.67
trees_j48	1,2,4,6,9	97.67	PCA 1,2,3,4,5,6,7,8	97.00

Column 1 lists different algorithms. Columns 2 & 4 list the best data subsets and Columns 3 & 5 accuracies, respectively. (1: Pfam; 2: Orthology; 3: Prot_interactions; 4: Best Blast hits; 5: Subcellular localization; 6: Functional linkages; 7: HPs linked to Pseudogenes 8: Homology modelling; 9: HPs linked to ncRNAs). Accuracies shown by both the subset combinations are almost same, with subset combinations from the complete dataset showing a slightly higher accuracy

Overall, the combined methods of feature selection provided ample evidence that all nine features are essential for a model generation. Correlation analysis has further allowed us to improve our classification feature selection pairs which tend to be positive for pfam and orthology (1 & 2); sub-cellular location and functional linkages (5 & 6); functional linkages and homology modelling (6 & 8) (detailed in Additional file 2). In addition, the two-tailed *p*-values for the above-mentioned combinations (1 & 2; 5 & 6; 5 & 8) were much less than the correlation (R) values, indicating that the association between those variables is statistically significant. We further analysed the performance of our model using various performance evaluation metrics which showed improved performance for the nine-point schema (Table 6, Additional file 3).

**Table 6:** Individual nine-point schema data are subjected through learning algorithms and scoring metrics are derived, averaged and tabulated. Values are compared with the six-point performance metrics

Algorithm	Sensitivity/ Recall (%)		Specificity (%)		Precision (%)		F_1_ Score (%)		MCC (%)	
	Six point	Nine point	Six point	Nine point	Six point	Nine point	Six point	Nine point	Six point	Nine point
Decision Tree (j48)	37	38	90	93	17	85	23	41	16	54
SVM (functions_smo_npolyk)	36	37	89	93	16	57	22	41	15	36
Neural networks(functions_mlp)	36	38	89	92	16	80	22	43	15	53
Naïve Bayes (Bayes_Naïve BayesUpdateable)	37	37	89	93	16	81	22	40	17	53

## Methods

### Construction of datasets

**Fig. 1:**
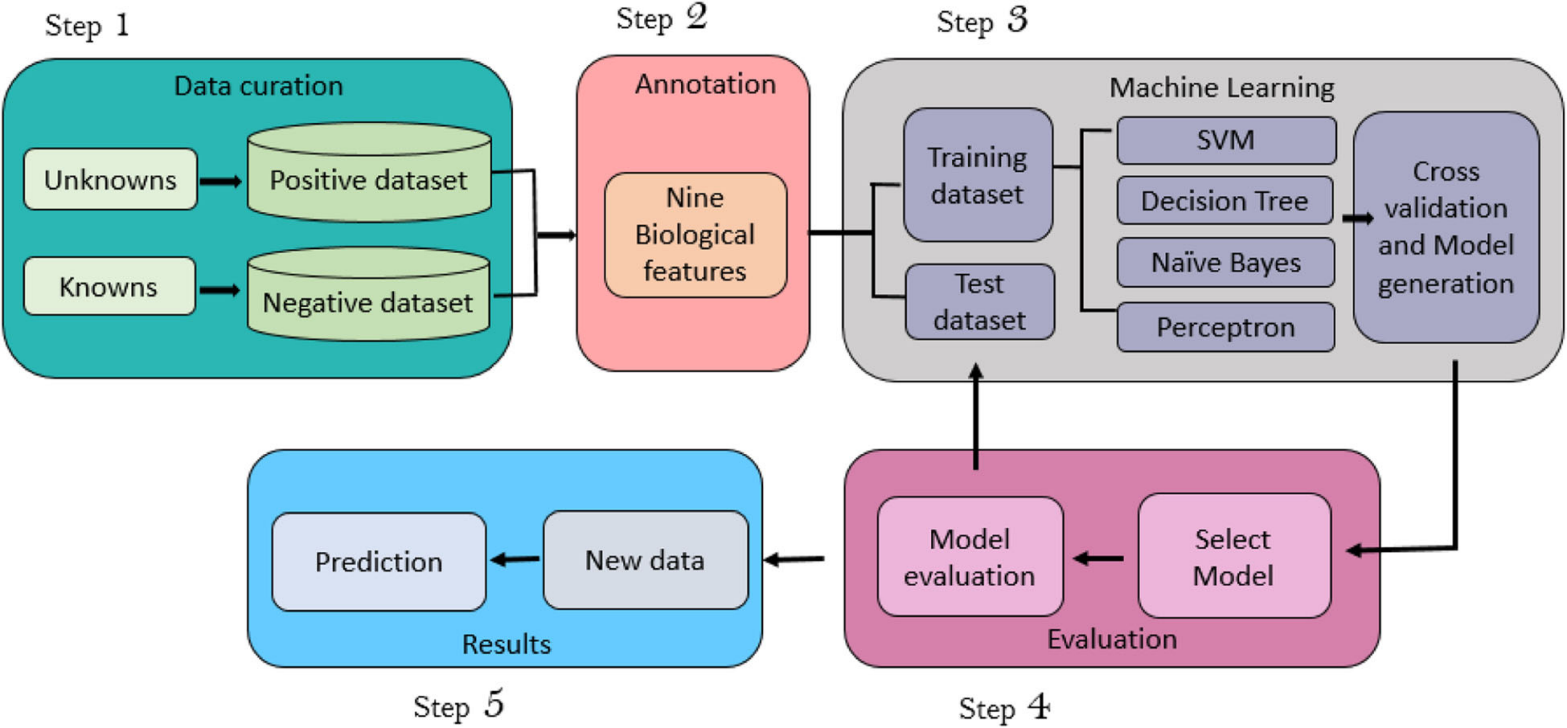
Methodology adopted to generate the classification model

Two datasets were prepared for this study, viz. positive and negative datasets, with the former constituting the HPs while the latter representing functional proteins. The final dataset consisted of 106 positive instances and 194 negative instances of HPs. These proteins were considered from GenBank with keyword searches “*Homo sapiens*” AND “Hypothetical Proteins” and further filtered with annotation across the tools (Additional file 4). The negative dataset was used to override false positives, thereby obtaining improved precision. Algorithms learn the characteristics underlying the known functional proteins from the given negative dataset. They are also used to validate the predicted results by making a comparison with known functional proteins. Finally, scores from all the nine classifiers were summed up to give total reliability score (TRS; Fig. 1).

### Significance of the features

The six features from our earlier proposed six-point classification scoring schema are pfam score, orthology inference, functional linkages, back-to-back orthology, subcellular location and protein associations taken from known databases and visualizers [31]. Conservation is one of the important features of essential proteins. Studies have proven that essential proteins evolve more slowly and are more evolutionarily conserved than non-essential proteins [32]. While we used sequence-based features like orthology, back-to-back orthology and domain analysis to describe the essentiality of the proteins from the perspective of evolutionary conservation [33], proteins often interact with each other to accomplish the biological functions of cells [34]. Apart from this, functional linkages [35] and subcellular localization [36] have been popular in predicting the essentiality or what we call the known-unknowns of proteins. Three new features that were considered in this model are HPs linked to pseudogenes, homology modelling and HPs linked to non-coding RNAs. Pseudogenes are the functionally deprecated sequences present in the genome of an organism. These disabled copies of genes are the products of gene duplication or retrotransposition of functional genes [37]. It is generally believed that the majority of the HPs are the products of pseudogenes [38]. This feature is employed to check if the HP is actually a pseudogene by performing tBLASTn, a variant of BLAST which considers proteins as a query and searches against the nucleotide database. The homology modelling feature was introduced to predict the essentiality of the protein based on the model generated. As the protein three-dimensional (3D) structure leads to function, there is a possibility to assign biological function to proteins, if one could generate the model to find any interacting domains through structural bioinformatics-based approaches [39]. Most of the HPs from GenBank lack protein-coding capacity. Similarly, non-coding RNAs by definition do not encode proteins. This indicates that some of the HPs may themselves be noncoding RNAs [40]. With this feature, we checked if HPs are associated with non-coding RNAs and are influenced by regulatory regions (detailed in Table 1).

### Classifier design and training

**Fig. 2:**
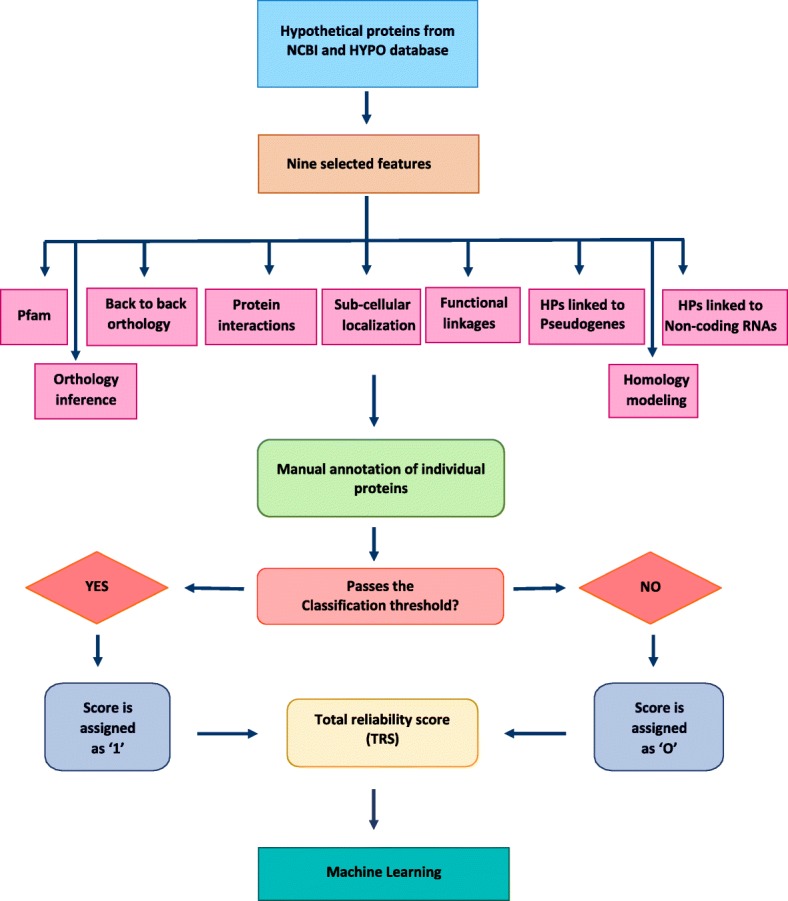
Workflow to annotate HPs across each classifier (Details in Additional file 2: Figure S1)

Prediction of the function of HPs can be presented as a binary classification problem. Each protein from both datasets was annotated across nine selected features and assigned a score of 1 if the protein met the criteria or 0 if it did not (Fig. 2). Criteria followed for scoring are shown in Additional file 5: Figure S1. The classifier was trained across the nine features according to the scores assigned to the members of each dataset. We used four major classifiers to train and test the model: (i) SVM (ii) Naïve Bayes (iii) Decision trees and (iv) Perceptron. For non-separable learning sets, a variant of perceptron called pocket algorithm [41] was used, which arbitrarily minimizes the error for the non-separable learning set [42]. It works by storing and using the best solution seen so far rather than relying on the last solution. These solutions appear purely stochastic. 80% of the dataset was used for training and the rest for testing. We performed 1000 independent iterations of SVM, Naïve Bayes and Perceptron algorithms. Instead of a k-fold cross-validation, we considered 1000 independent iterations and averaged their results so as to avoid over-fitting, assuming that a *k* for such a problem is beyond the scope of this work. Further, we analysed the data using the Waikato Environment for Knowledge Analysis (WEKA) software package (version 3.8) [43] where 37 other learning algorithms were used along with the aforementioned four major algorithms. WEKA was implemented for classifier design, training and evaluation. Finally, Jaccard indices followed by training the datasets using machine learning algorithms were used to infer heuristics.

### Performance evaluation

Evaluating the performance of learning algorithms is a central aspect of machine learning. Several measures including cross-validation as a standard method [44] and a 10-fold cross-validation using WEKA were applied to test the performance of the predictive model. To mitigate the over-fitting problem, the following measures were used to evaluate the performance of the classifiers: accuracy, sensitivity, specificity, F_1_ score, Matthew’s Correlation Coefficient (MCC) [45, 46]. Specificity, Precision, Sensitivity and MCC of 1 indicate perfect prediction accuracy [47].

The measures are defined as follows:

Accuracy = (TP + TN) / (TP + FN + FP + TN).

Sensitivity (Recall) = TP / (TP + FN).

Specificity = TN / (TN + FP).

Precision = TP / (TP + FP).

F_1_ Score = 2(Precision * Recall) / (Precision + Recall).

Matthews Correlation Coefficient (MCC).

= ((TP x TN) - (FP x FN)) / (TP + FP) (TP + FN) (TN + FP) (TN + FN).

where TP: True Positives (positive samples classified correctly as positive), TN: True Negatives (negative samples classified correctly as negative), FP: False Positives (negative samples predicted wrongly as positive) and FN: False Negatives (positive samples predicted wrongly as negative).

## Conclusion

We have proposed a nine-point classification scoring schema to help functionally annotate the HPs. While a large number of heuristics were interpreted to introduce such problems, there is a strong need to ensure that the HPs in question are provided a function in silico. An attempt has been made to close the gap of providing functional linkages to HPs. The addition of classification features would possibly serve as a valuable resource for analysing data and for understanding the known-unknown regions. The potential regulatory function of HPs could be determined if there are larger curated datasets. However, this is also influenced by how the HPs interact with each other, given a new set of dimensions in the form of next-generation sequencing to the scientific community.

## Additional files

Additional file 1: **Figure S2.** Jaccard index plot showing the coefficient distances for the HPs. The x-axis indicates the HPs while the y-axis indicates the distance. (XLSX 20 kb)

Additional file 2: Tables showing correlation analysis (XLSX 150 kb)

Additional file 3: Learning algorithms results (XLSX 11 kb)

Additional file 4: List of HPs which we used for classification and machine learning approaches (XLSX 23 kb)

Additional file 5: **Figure S1.** Workflow adopted for annotation and scoring of HPs across each classifier (PDF 249 kb)

Additional file 6: Performance evaluation (PDF 152 kb)

## Abbreviations

BLAST: Basic local alignment search tool
HP: Hypothetical protein
WEKA: Waikato environment for knowledge analysis

## References

[1] Uhlen M (2010). Towards a knowledge-based human protein atlas. Nat Biotechnol.

[2] Galperin MY (2001). Conserved ‘hypothetical’ proteins: new hints and new puzzles. Comp Funct Genomics.

[3] Nimrod G (2008). Detection of functionally important regions in “hypothetical proteins” of known structure. Structure.

[4] Shahbaaz M (2013). Functional annotation of conserved hypothetical proteins from *Haemophilus influenzae* Rd KW20. PLoS One.

[5] Mohan R, Venugopal S (2012). Computational structures and functional analysis of hypothetical proteins of Staphylococcus aureus. Bioinformation.

[6] Murakami M (2005). InCeP: intracellular pathway based on mKIAA protein-protein interactions. DNA Res.

[7] Ijaq J (2015). Annotation and curation of uncharacterized proteins-challenges. Front Genet.

[8] Shoemaker BA, Panchenko AR (2007). Deciphering protein–protein interactions. Part I. Experimental techniques and databases. PLoS Comp Biol.

[9] Zhang LV (2004). Predicting co-complexed protein pairs using genomic and proteomic data integration. BMC Bioinformatics.

[10] Ji Y (2001). Identification of critical staphylococcal genes using conditional phenotypes generated by antisense RNA. Science.

[11] Kamath RS (2003). Systematic functional analysis of the *Caenorhabditis elegans* genome using RNAi. Nature.

[12] Giaever G (2002). Functional profiling of the *Saccharomyces cerevisiae* genome. Nature.

[13] Gallagher LA (2007). A comprehensive transposon mutant library of Francisella novicida, a bioweapon surrogate. Proc Natl Acad Sci.

[14] Enright AJ (1999). Protein interaction maps for complete genomes based on gene fusion events. Nature.

[15] Sivashankari S, Shanmughavel P (2006). Functional annotation of hypothetical proteins-a review. Bioinformation.

[16] Lei X (2018). Predicting essential proteins based on RNA-Seq, subcellular localization and GO annotation datasets. Knowl-Based Syst.

[17] Li M (2018). Identifying essential proteins based on sub-network partition and prioritization by integrating subcellular localization information. J Theor Biol.

[18] Sperschneider J (2017). LOCALIZER: subcellular localization prediction of both plant and effector proteins in the plant cell. Sci Rep.

[19] Zhen C (2018). The lncLocator: a subcellular localization predictor for long non-coding RNAs based on a stacked ensemble classifier. Bioinformatics.

[20] Eisen MB (1998). Cluster analysis and display of genome-wide expression patterns. Proc Natl Acad Sci U S A.

[21] Deng M (2003). Prediction of protein function using protein-protein interaction data. J Comput Biol.

[22] Bock JR, Gough DA (2001). Predicting protein-protein interactions from primary structure. Bioinformatics.

[23] Fariselli P (2002). Prediction of protein--protein interaction sites in heterocomplexes with neural networks. Eur J Biochem.

[24] Troyanskaya OG (2003). A Bayesian framework for combining heterogeneous data sources for gene function prediction (in *Saccharomyces cerevisiae*). Proc Natl Acad Sci U S A.

[25] Jansen R (2003). A Bayesian networks approach for predicting protein–protein interactions from genomic data. Science.

[26] Chen XW, Liu M (2005). Prediction of protein–protein interactions using random decision forest framework. Bioinformatics.

[27] Marcotte EM (1999). Detecting protein function and protein–protein interactions from genome sequences. Science.

[28] Nigatu D, Henkel W (2017). Prediction of essential genes based on machine learning and information theoretic features.

[29] Li M (2017). United complex centrality for identification of essential proteins from PPI networks. IEEE/ACM Trans Comput Biol Bioinform.

[30] You Z-H (2017). Highly efficient framework for predicting interactions between proteins. IEEE Trans Cybern.

[31] Suravajhala P, Sundararajan VS (2012). A classification scoring schema to validate protein interactors. Bioinformation.

[32] Gustafson AM (2006). Towards the identification of essential genes using targeted genome sequencing and comparative analysis. BMC Genomics.

[33] Deng J (2010). Investigating the predictability of essential genes across distantly related organisms using an integrative approach. Nucleic Acids Res.

[34] Peng W (2012). Iteration method for predicting essential proteins based on orthology and protein-protein interaction networks. BMC Syst Biol.

[35] Wang J (2013). Computational approaches to predicting essential proteins: a survey. Proteomics Clin Appl.

[36] Li G (2016). Predicting essential proteins based on subcellular localization, orthology and PPI networks. BMC Bioinformatics.

[37] Mighell AJ (2000). Vertebrate pseudogenes. FEBS Lett.

[38] Shidhi PR (2014). Identifying pseudogenes from hypothetical proteins for making synthetic proteins. Syst Synth Biol.

[39] França TC (2015). Homology modeling: an important tool for the drug discovery. J Biomol Struct Dyn.

[40] Jia H (2010). Genome-wide computational identification and manual annotation of human long noncoding RNA genes. RNA.

[41] Gallant SI (1990). Perceptron-based learning algorithms. IEEE Trans Neural Netw.

[42] Muselli M (1997). On the convergence properties of the pocket algorithm. IEEE Trans Neural Netw.

[43] Frank E (2016). The WEKA Workbench. Online Appendix for “Data Mining: Practical Machine Learning Tools and Techniques”, Morgan Kaufmann, Fourth Edition.

[44] Hu P (2007). Computational prediction of cancer-gene function. Nature Rev Cancer.

[45] Baldi P (2000). Assessing the accuracy of prediction algorithms for classification: an overview. Bioinformatics.

[46] Matthews BW (1975). Comparison of the predicted and observed secondary structure of T4 phage lysozyme. Biochim Biophys Acta.

[47] Saito T, Rehmsmeier M (2015). The precision-recall plot is more informative than the ROC plot when evaluating binary classifiers on imbalanced datasets. PLoS One.

