# Supplementary material for: A model to predict the function of hypothetical proteins through a nine-point classification scoring schema
Source: BMC Bioinformatics. 2019 Jan 8;20:14. doi: 10.1186/s12859-018-2554-y (PMC6325861; doi:10.1186/s12859-018-2554-y)
Supplement: Supplementary file 5 — Figure S1. Workflow adopted for annotation and scoring of HPs across each classifier (PDF 249 kb) [file 12859_2018_2554_MOESM5_ESM.pdf]

Putative gene: From Uniprot

Classifier 1: Pfam

1. Protein sequence in FASTA format from NCBI
2. Pfam database
3. Sequence search
4. Results recorded

Scoring: Protein meet the classifier- 1, else 0. E-value: <1 = 0; >1 or near to 1 = 0

Classifier 2: Orthology

1. Protein sequence in FASTA format from NCBI
2. BLAST
3. Best hit recorded

Scoring: E value: <1 =1; >1 =0

Classifier 3: Protein interactions/Association studies

1. Protein sequence in FASTA format from NCBI
2. STRING, BioGRID
3. Search
4. Organism - Homo sapiens
5. Selected best interactors
6. Selection criteria: Score & experimental evidence
7. Method: Experimental evidence

Scoring: If I Could fInd same interactors in atleast two databases, score = 1, else 0.

*BioGrid and STRING are given priority

Classifier 4: Bidirectional best blast hits (BBH)

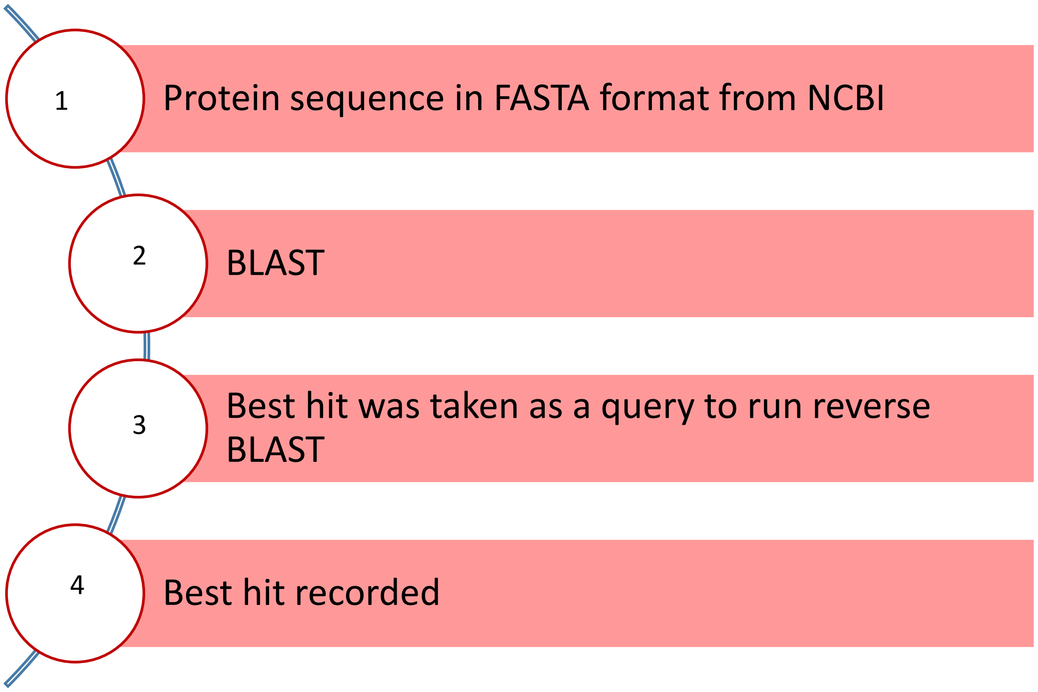

Scoring: If reverse blast of protein A resulted in protein A as a best hit (BBH), gave score as 1, else 0

Protein A - - - -BLAST- - - - Best hit - - - REVERSE BLAST - - - protein A - 1

Protein A - - - -BLAST- - - - Best hit - - - REVERSE BLAST - - - protein B - 0

Classifier 5: Sorting signals

1. Protein sequence in FASTA format from NCBI
2. Searched against TargetP, WoLF PSORT, ESLpred
3. TargetP: Organism group- Non-plant; Cutoffs -default
4. WoLF PSORT: Organism - Animal
5. ESLpred: Prediction approach - Hybrid approach based
6. Localization selected based on the scores
7. Consensus

Scoring: Could come to consensus from three databases, score =1, else 0

For ESLpred, order of priority: Nuclear > Cytoplasm > Mitochondria > Extracellular

Classification 6: Functional linkages from known databases

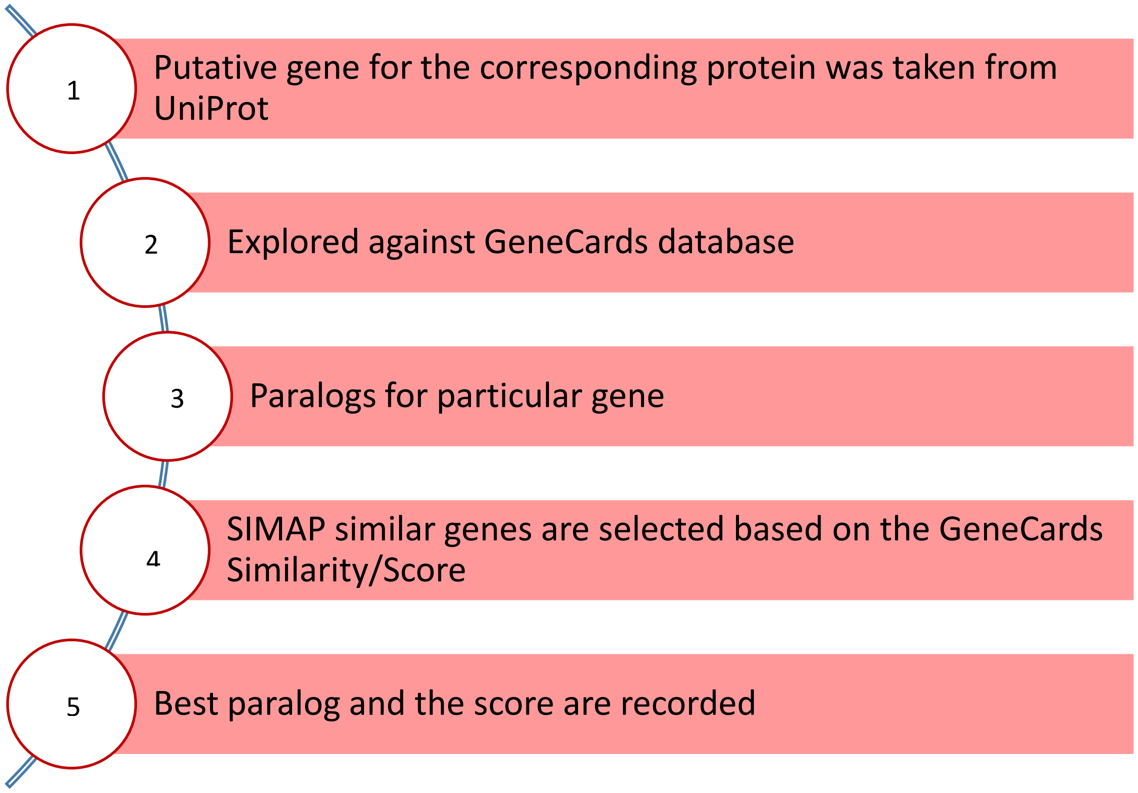

Scoring: If paralog was found, score = 1, if not 0

Classification 7: HPs linked to Pseudogenes

1. Bidirectional best blast hit was taken as a query
2. FASTA sequence was selected
3. tblastn
4. Sequences not starting with Methionine are selected
5. Criteria: Homo sapiens; %identity, E-value
6. Accessions numbers are recorded

Scoring: 1. Predicted and synthetic sequences are ignored.

2. Sequences from Homo sapiens are considered.

3. E- value less than zero is considered

4. No end to end alignment

Sequences not starting with Methionine and meeting all the above criteria, given score 1, else 0.

Classification 8: Homology modelling

1. Protein sequence in FASTA format from NCBI
2. Position specific iterated BLAST (PSI-BLAST) was carried against Protein Data Bank
3. Homologous experimental structures were identified
4. 3D structures were modelled using Swiss-Model server
5. PDB template, its description and % identity were recorded

Scoring: Based on % Identity between query and PDB template

If there is more than 30% similarity, score =1, else 0.

Classification 9: HPs linked to Non-coding RNAs

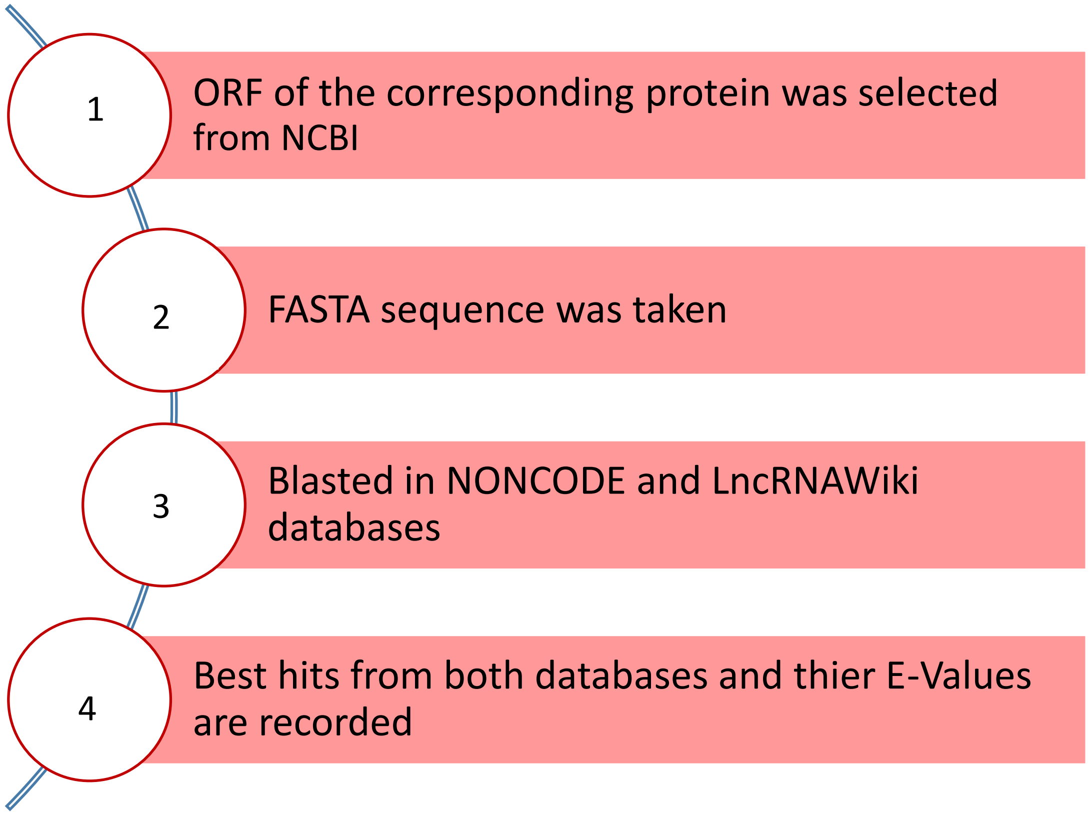

Scoring: Only sequences from Homo sapiens are considered (NONCODE)

<u>Criteria for scoring</u>

H. sapiens sequence in top three hits

Top five hits are considered when there is no considerable difference between scores of first five hits.

E value less than zero

If the above criteria are met, score 1, else 0.
