# Supplementary material for: A model to predict the function of hypothetical proteins through a nine-point classification scoring schema
Source: BMC Bioinformatics. 2019 Jan 8;20:14. doi: 10.1186/s12859-018-2554-y (PMC6325861; doi:10.1186/s12859-018-2554-y)
Supplement: Supplementary file 6 — Performance evaluation (PDF 152 kb) [file 12859_2018_2554_MOESM6_ESM.pdf]

S.No.	Algorithm	TP Rate	FP Rate	Precision	Recall	F-measure	MCC	ROC Area	PRC Area
1	NaiveBayes_MultinomialUpdateable	0.374	0.187	0.170	0.374	0.231	0.157	0.437	0.219
2	NaiveBayes_Updateable	0.369	0.172	0.161	0.369	0.222	0.167	0.507	0.218
3	functions_mlp	0.360	0.178	0.162	0.360	0.221	0.151	0.499	0.215
4	SimpleLogistic	0.369	0.182	0.162	0.369	0.223	0.158	0.510	0.213
5	smo_npolyk	0.365	0.183	0.164	0.365	0.225	0.151	0.583	0.265
6	smo_PolyK	0.365	0.183	0.164	0.365	0.225	0.151	0.583	0.265
7	smo_Puk	0.365	0.183	0.164	0.365	0.225	0.151	0.583	0.265
8	smo_RBFK	0.365	0.183	0.164	0.365	0.225	0.151	0.583	0.265
9	HoeffdingTree	0.342	0.163	0.154	0.342	0.205	0.150	0.514	0.223
10	Lazy_IBK	0.383	0.175	0.181	0.383	0.241	0.178	0.516	0.228
11	LogisticRegression	0.374	0.178	0.168	0.374	0.230	0.165	0.501	0.211
12	misc_InputMappedClassifier	0.243	0.243	0.059	0.243	0.095	0.000	0.290	0.167
13	rules_DecisionTable	0.333	0.202	0.155	0.333	0.190	0.124	0.480	0.221
14	rules_JRIP	0.243	0.243	0.060	0.243	0.096	0.001	0.297	0.170
15	rules_OneR	0.387	0.175	0.178	0.387	0.241	0.184	0.607	0.253
16	rules_PART	0.374	0.177	0.168	0.374	0.230	0.164	0.512	0.237
17	rules_ZeroR	0.243	0.243	0.059	0.243	0.095	0.000	0.290	0.167
18	trees_DecisionStump	0.374	0.179	0.167	0.374	0.229	0.164	0.502	0.230
19	trees_j48	0.374	0.177	0.168	0.374	0.230	0.164	0.512	0.237
20	trees_RandomForest	0.369	0.176	0.167	0.369	0.227	0.158	0.518	0.218
21	trees_LMT	0.369	0.182	0.162	0.369	0.223	0.158	0.510	0.213
22	trees_RandomTree	0.383	0.175	0.181	0.383	0.241	0.178	0.510	0.234
23	trees_REPTree	0.356	0.189	0.156	0.356	0.212	0.145	0.517	0.236

S.No.	Algorithm	Specificity
1	NaiveBayes_Updateable	0.898
2	smo_npolyk	0.895
3	functions_mlp	0.894
4	J48	0.899
